# Rapid Construction of an Infectious Clone of Fowl Adenovirus Serotype 4 Isolate

**DOI:** 10.3390/v15081657

**Published:** 2023-07-29

**Authors:** Minzhi Gong, Yating Wang, Shijia Liu, Boshuo Li, Enqi Du, Yupeng Gao

**Affiliations:** 1 College of Veterinary Medicine, Northwest A&F University, Yangling 712100, China; gongminzhi1160@nwsuaf.edu.cn (M.G.); yating@nwafu.edu.cn (Y.W.); liushijia1995@163.com (S.L.); liboshuo2021@163.com (B.L.); 2 Yangling Carey Biotechnology Co., Ltd., Yangling 712100, China

**Keywords:** FAdV-4, infectious clone, IIIa gene, SC-Ad vectors, recombinant adenovirus vectors

## Abstract

Adenovirus vectors possess a good safety profile, an extensive genome, a range of host cells, high viral yield, and the ability to elicit broad humoral and cellular immune responses. Adenovirus vectors are widely used in infectious disease research for future vaccine development and gene therapy. In this study, we obtained a fowl adenovirus serotype 4 (FAdV-4) isolate from sick chickens with hepatitis–hydropericardium syndrome (HHS) and conducted animal regression text to clarify biological pathology. We amplified the transfer vector and extracted viral genomic DNA from infected LMH cells, then recombined the mixtures via the Gibson assembly method in vitro and electroporated them into EZ10 competent cells to construct the FAdV-4 infectious clone. The infectious clones were successfully rescued in LMH cells within 15 days of transfection. The typical cytopathic effect (CPE) and propagation titer of FAdV-4 infectious clones were also similar to those for wild-type FAdV-4. To further construct the single-cycle adenovirus (SC-Ad) vector, we constructed SC-Ad vectors by deleting the gene for IIIa capsid cement protein. The FAdV4 infectious clone vector was introduced into the ccdB cm expression cassette to replace the IIIa gene using a λ-red homologous recombination technique, and then the ccdB cm expression cassette was excised by *Pme*I digestion and self-ligation to obtain the resulting plasmids as SC-Ad vectors.

## 1. Introduction

Fowl adenovirus (FAdV) belongs to the Aviadenovirus genus in the family Adenoviridae. FAdV can be grouped into five species (FAdV-A to FAdV-E) based on their molecular structure, and 12 serotypes (FAdV-1 to 8a and 8b to 11) have been identified based on their serological relationships [1,2]. Fowl adenovirus serotype 4 (FAdV-4) has been grouped with the FAdV-C species; it is highly pathogenic to chickens, particularly 3–5-week-old broilers. It is a double-stranded DNA virus with a genome of approximately 45 kb and encodes 10 major structural proteins and 11 nonstructural proteins [3,4,5,6]. FAdV-4 causes hydropericardium syndrome (HPS), inclusion body hepatitis (IBH), respiratory tract disease, and gizzard erosion in chickens [7,8,9,10,11]. Severe disease in broiler chickens is characterized by the accumulation of clear, straw-colored fluid in the pericardial sac, as well as nephritis and hepatitis [12,13]. Since 2015, outbreaks of novel FAdV-4 genotype infections have led to considerable financial losses in the poultry sector in China. 

Given their strong gene expression, high titers, and ability to induce an immunological response, adenoviral (Ad) vectors have demonstrated potential as vaccine platforms. [14,15]. With the advancements of reverse genetic technology and gene editing, several viruses have been successfully modified into viral vectors, including adenovirus [16], pox- virus [17], etc. Adenovirus vectors are widely applied in gene therapy [18], as well as in vaccine development against emergent viruses such as SARS-CoV-2 [19], MERS-CoV [20], and Ebola [21]. There is a lot of research on the fowl adenovirus vector, which can also insert and express protective antigen genes of pathogens for vaccine development [22,23,24,25]. In previous studies, fowl adenovirus FAdV-9 infectious clones have proven valuable for research into the in vitro growth characteristics, the function of viral genes, and the discovery of non-essential areas for virus engineering [26,27,28].

Adenovirus can be designed by directly operating viral genomes in vitro or by using infectious clones. Directly manipulating the viral genome is cumbersome and time-consuming; on the contrary, infectious clones can be readily controlled and manufactured on a massive scale. The construction of an Ad vector is challenging as the genome is very large and has few restriction sites available. Classical methods are based on homologous recombination or rely on rare restriction sites; again, these methods are time-consuming and difficult to control [29,30]. Gibson DNA assembly technology, first proposed by Daniel G. Gibson and his colleagues in 2009, is a recombination-based molecular cloning method for the in vitro assembly of DNA fragments [31]. The methodology enables the easy assembly of multiple DNA fragments into a circular plasmid in a single-tube isothermal reaction. Since the ligation reaction does not depend on the restriction enzyme cleavage site and can connect multiple DNA fragments simultaneously, this technology has advantages over traditional enzyme digestion ligation technology and is seeing increasing use [32,33]. Here, we have successfully utilized Gibson DNA Assembly technology to construct infectious clones of wild-type FAdV-4.

Currently, the majority of Ad vectors are E1-deleted Ad vectors. E1 gene-deleted replication-defective Ad (RD-Ad) vectors are relatively safe; however, there are fewer copies of the viral genome due to lower transgene protein production, which can lead to weak transgene-directed immune responses. Replication-competent Ad (RC-Ad) vectors may mediate robust transgene expression but can create adenovirus disease risk in vaccine recipients. In 2014, a novel adenovirus vector named single-cycle Ad (SC-Ad) was reported, which can replicate its genome and transgene, but avoid the risk of causing adenovirus infections by deleting the capsid cement protein IIIa [34]. When the IIIa gene is deleted, mature virions are not formed, and the viral DNA is not packaged, but these phenotypes can be rescued in IIIa-expressing cells, resulting in higher viral genome copy numbers to increase transgene expression and enhancing immune responses in a manner directly proportional to the number of infectious virions [34,35]. In this study, to acquire the capacity to reproduce transgenes while avoiding the dangers associated with utilizing fully replicated Ad vectors, we created SC-Ad by removing the IIIa gene from infectious clones of FAdV-4.

## 2. Materials and Methods

### 2.1. Plasmid and Cell

LMH cells (ATCC CRL-2117) were cultured with DMEM medium containing 10% fetal bovine serum (FBS) at 37 °C in a 5% CO_2_ incubator. pFPAV3 and pKD46 were preserved in our laboratory.

### 2.2. Virus Isolation

During an HHS epidemic at a poultry farm in Shaanxi Province, China, liver samples were collected from a deceased chicken. Samples were mixed with phosphate-buffered saline (PBS), sheared, and ground into a liquid form. The viral solution was subjected to three rounds of the freeze-thaw procedure and was centrifuged at 4 °C, 15,292× *g* for 10 min. The viral solution was filtered with a 0.22 μm microporous filter and inoculated into 6–8-day-old SPF chicken embryos incubated in a 37 °C incubator. The embryos were candled every 12 h and dead embryos discarded within 24 h. After 9 days, allantoic fluid was extracted. The freeze-thawing of allantoic fluid was repeated 3 times, followed by centrifugation to remove cell debris: 15,292× *g* for 10 min. The above allantoic fluid was blindly passed on embryonated chicken eggs three times. The allantoic fluid was used as the seed virus to infect LMH cells incubated at 37 °C in 5% CO_2_. The cytopathic effect (CPE) of LMH cells was observed at 36, 72, and 96 h after infection. The culture was freeze-thawed in three rounds, cycling from 37 °C to −80 °C. To remove cell debris, centrifugation was performed at 15,292× *g* for 10 min, and the supernatant was cryopreserved at −80 °C.

### 2.3. PCR Amplification and Sequencing of the Hexon Gene

Utilizing a modified version of the traditional genome extraction approach, adenoviral genomic DNA was recovered from viral culture [36] and used as a template to amplify the partial hexon gene sequence of FAdV-4 via PCR using specific hexon-F and hexon-R primers [37] (Table A1). The amplified product was visualized using 1% agarose gel electrophoresis and purified using the Gel Extraction Kit (TIANquick Midi Purification Kit, Beijing, China). The purified PCR products were ligated into a pMD19-T plasmid, which was transformed into Top10 competent cells. The cells were spread on ampicillin-containing LB agar plates and incubated at 37 °C for 12 h. The positive colonies were picked and grown in ampicillin-containing liquid LB medium at 37 °C with shaking for 6 h, validated by colony PCR, and subjected to direct Sanger sequencing by Beijing Tsingke Biotech Co., Ltd. (Beijing, China). 

### 2.4. Hexon Gene Sequence Analysis and Phylogenetic

The nucleotide sequence of the hexon gene of the FAdV isolate was compiled using SnapGene. The 18 reference strains of FAdV were retrieved from the NCBI GenBank database. The phylogenetic tree was built using the neighbor-joining method by MEGA 7.0.

### 2.5. Animal Regression Test

Twenty chicks hatched from the eggs of white specific pathogen-free (SPF) broilers acquired from the Animal Experimental Centre of Yangling Lvfang Biological Engineering Co., Ltd., Yangling, China, were raised in an isolator. At twenty days of age, ten of these chicks were intramuscularly injected with third-passage viruses from LMH cells (0.2 mL per chicken, virus titer 1 × 10^7^ TCID_50_/mL) maintained in LMH cells, and the other chicks were intramuscularly injected with the same dose of physiological saline for use as the negative control. Clinical symptoms and pathological changes were observed and recorded 3 times per day. The dead chickens were quickly dissected, and pathological changes in heart and liver tissue were observed. After 14 days of continuous observation, the surviving chickens were dissected, and the results were recorded. 

### 2.6. Construction of the Recombinant pShuttle-FAdV-4 Plasmid with the DNA Assembly Technique

First, using the swine adenovirus type 3 infectious clone(pFPAV3) as a template, the primers (pShuttle-*Pac*I-ITR-F/pShuttle-*Pac*I-ITR-R) (Table A1) flanked by the partial inverted terminal repeat (ITR) sequence of FAdV-4 were synthesized to amplify a plasmid backbone containing pShuttle-FAdVITR-FR. Simultaneously, *Pac*I and *I-Sce*I restriction sites were integrated into the ITR sequence. PCR products were detected via 1% agarose gel electrophoresis and purified using the Gel Extraction Kit (TIANquick Midi Purification Kit, Beijing, China).

We used the Gibson DNA assembly method to create fowl adenovirus vectors. Using the NEBuildER HiFi DNA Assembly Master Mix (Cat. E2623S, New England Biolabs, Ipswich, MA, USA), 200 ng of pShuttle-FAdVITR-FR plasmid backbone and 1 μg of FAdV-4 genome were mixed, and the mixtures were incubated at 50 °C for 60 min in a thermocycler. One microliter of the assembled product was used to transform EZ10 competent cells. The cells were spread on kanamycin-containing LB agar plates and incubated at 37 °C overnight. 

The positive colonies were picked and grown in liquid LB medium containing kanamycin at 37 °C overnight with shaking. Colony PCR was performed with the primers FAdV4-23500-F/FAdV4-24501-R and FAdV4-ITR-F/FAdV4-1176-R (Table A1). The products were detected on a 1% agarose gel, and the plasmids were extracted. The plasmids carrying the FAdV-4 genome were named pShuttle-FAdV-4. The plasmids of consistent size in colonies were digested with *Bam*HI and *Pac*I, visualized on a 1% agarose gel, extracted, and confirmed to contain the viral genome. 

### 2.7. Rescue of FAdV-4 Infectious Clones 

To rescue FAdV-4 infectious clones, 80% confluent monolayer of LMH cells in 6-well plates were transfected overnight with Lipofectamine 3000 (Thermo Fisher Scientific-CN, Shanghai, China) with 5 μg of the *Pac*I-linearized adenovirus plasmid pShuttle-FAdV-4 including genome (recovered via ethanol precipitation). Then, the medium was changed to DMEM plus 2% FBS. When the CPE reached 80%, the culture was harvested. The culture was freeze-thawed for three rounds and centrifuged at 15,292× *g* for 10 min to remove cell debris. The suspensions were named rFAdV-4 (P0 generation). The viral titer was detected via TCID_50_, and the viruses were serially passaged in LMH cell culture until a full cytopathic effect occurred. The recombinant adenoviruses were passaged for 3 generations (P3).

### 2.8. Construction of Replicating Single-Cycle FAdV-4 Vector

Homologous recombination was used to knock out the pIIIa gene with positive and negative screening markers. The thermal-sensitive pKD46 plasmid with λ-red homologous recombinase was transformed through electroporation into competent pShuttle-FAdV-4/DB3.1 cells. After being evenly distributed on LB agar plates containing kanamycin, the cells were cultured for one day at 30 °C. The pKD46 and pShuttle-FAdV-4/DB3.1 positive colonies were selected and cultivated in liquid LB medium with kanamycin and ampicillin at 30 °C with shaking at 220 r/min. Cell concentration was determined by measuring absorption at OD_600nm_. L-arabinose was added to induce the expression of the homologous recombination system encoded by the plasmid pKD46.

The pKD46 and pShuttle-FAdV-4/DB3.1 competent cells were transformed together with the donor of the entire ccdB cm expression cassette via electroporation. The cells were spread on LB agar plates containing kanamycin, ampicillin, and chloramphenicol and incubated at 30 °C overnight.

Positive colonies (pKD46 and pShuttle-FAdV-4(ΔpIIIa:ccdB-Cm)/DB3.1) were collected and cultured in liquid LB medium containing ampicillin and kanamycin at 30 °C; this was confirmed by colony PCR with the primers IIIa-up-300-F/IIIa-down-200-R (Table A1).

The plasmid pKD46 was incubated at 42 °C for two cycles to remove pKD46. After removing the pKD46 plasmid, the pShuttle-FAdV-4(ΔpIIIa:ccdB-Cm)/DB3.1 colonies were confirmed by streak cultivation on kanamycin and ampicillin-containing LB agar plates. The pKD46 eliminated strain was named pShuttle-FAdV-4(ΔIIIa:ccdB-Cm)/DB3.1. 

The plasmids were further linearized via *Pme*I digestion, and ccdB cm was isolated from agarose gel, self-ligated, and transformed into EZ10 competent cells for screening to obtain pShuttle-FAdV-4(ΔIIIa:*Pme*I)/EZ10, after which the IIIa gene was deleted. The deletion of IIIa gene-positive colonies was validated by colony PCR with IIIa-up-300-F/IIIa-down-200-R primers.

## 3. Results

### 3.1. Virus Isolation and Identification

**Figure 1:**
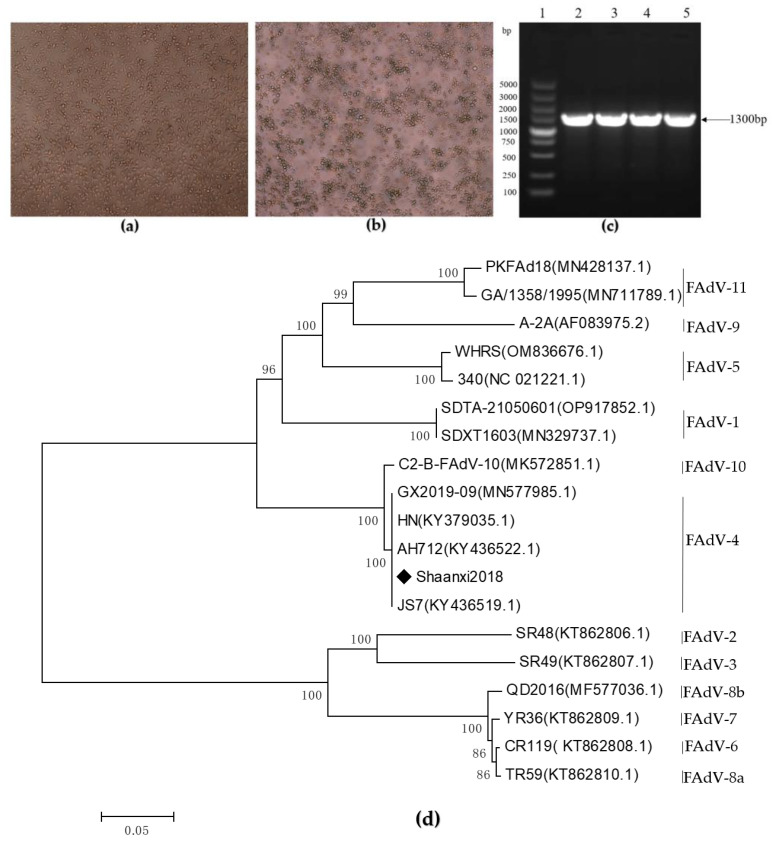
Virus isolation and identification. (**a**) Normal LMH cells, (**b**) the cytopathic effect (CPE) in LMH cells at 72 h post-transfection. (**c**) PCR amplification of hexon gene fragment. Lane1: DL5000 DNA Marker; Lane 2–5: amplify the product of the hexon gene. (**d**) Hexon-gene-based phylogenetic analysis. The tree was constructed using MEGA 7.0 software according to the neighbor-joining method (1000 replicates for bootstrap). ◆ represents the isolate in this study.

The adenovirus was isolated and identified through the inoculation of SPF chicken embryo with homogenized suspected tissues, and the chicken embryos were then inoculated into LMH cells. Normal LMH cells showed plum-blossom-like growth (Figure 1a). CPE of LMH cells was observed post-transfection during incubation at 37 °C in a humidified atmosphere supplemented with 5% CO_2_. At 72 h, typical CPE was observed (Figure 1b). Using viral culture supernatant DNA as templates, PCR amplification yielded a specific fragment of the hexon gene (Figure 1c). The amplification product of the hexon gene was recovered and cloned into the T vector pMD19-T, which was successfully constructed by sequencing verification. The hexon sequence was aligned with fowl adenovirus reference sequences from the GenBank database using ClustalW and classified according to standard taxonomic classifications. A phylogenetic tree was constructed using the neighbor-joining method, using the p-distance method (on 1000 bootstrapped datasets), revealing that the wild strain (Shaanxi2018) belonged to the serotype FAdV-4 (Figure 1d). The isolate was located in the same branch as some of the FAdV-4 reference strains, which showed higher homology and closer affinity.

### 3.2. Animal Regression Test

**Figure 2:**
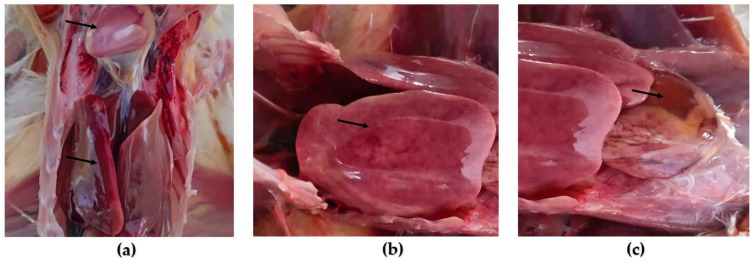
Gross lesions of chickens infected with FAdV-4. (**a**) Negative control: A normal liver and heart. (**b**) Challenge group: Pathological changes in the liver. (**c**) Challenge group: Pathological changes in the heart. The arrows in the figure refer to a normal or diseased liver or heart.

At 24 h after 10 chicks were intramuscularly injected with the P3-generation virus, huddling, chills, and depression were observed, and food intake decreased. After 72 h, two chicks died, with a 20% mortality rate, which corresponded to the clinical symptoms and histopathology of natural infections. No histopathological lesions were observed in the negative control group (Figure 2a). However, acute necrotic hepatitis with hemorrhagic, friable liver, hemorrhagic, swollen and yellowish (Figure 2b), and pericardial effusion, in which a clear straw-colored effusion is visible in the pericardial sac (Figure 2c).

### 3.3. Construction of FAdV-4 Infectious Clones 

**Figure 3:**
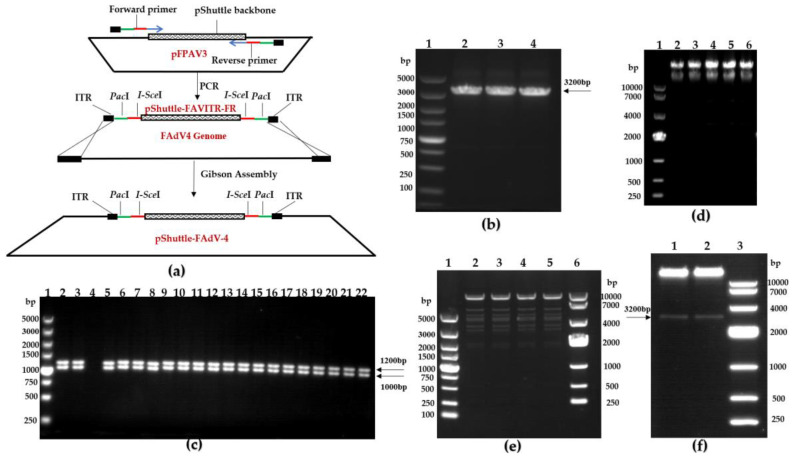
Construction of FAdV-4 infectious clones. (**a**) Schematic diagram of the construction. (**b**) Amplification of the plasmid backbone pShuttle-FAVITR-FR. Lane1: DL5000 DNA Marker; Lane 2–4: PCR fragment pShuttle-FAVITR-FR. (**c**) Identification of infectious clones of FAdV-4 by PCR. Lane 1: DL5000 DNA Marker; Lane 2–22: identified colonies. (**d**) Extraction of pShuttle-FAdV-4 plasmid. Lane 1: DL10000 DNA Marker; Lane 2: pFPAV3 plasmid; Lane 3–6: pShuttle-FAdV-4 recombinant plasmids. (**e**) *Bam*HI restriction enzyme analysis of recombinant plasmids pShuttle-FAdV-4. Lane 1: DL5000 DNA Marker; Lane 2–5: digestion of recombinant plasmids pShuttle-FAdV-4; Lane 6: DL10000 DNA Marker. (**f**) *Pac*I digestion verification. Lane 1–2: digestion of recombinant plasmids pShuttle-FAdV-4; Lane3: DL10000 DNA Marker.

The pShuttle-FAdV-4 plasmid was created according to the schematic diagram shown below (Figure 3a). First, the plasmid backbone pShuttle-FAdVITR-FR was amplified by pShuttle-*Pac*I-ITR-F/pShuttl*e-Pac*I-ITR-R primers. The amplified products were detected in a 1% agarose gel, revealing an approximately 3 kb fragment (Figure 3b). The FAdV-4 genome was then assembled into the pShuttle-FAdVITR-FR plasmid backbone. Two pairs of primers (FAV4-23500-F/FAV4-24501-R and FAV4-ITR-F/FAV4-1176-R) were used for colony PCR to determine the size of amplified fragments. PCR products were analyzed on a 1% agarose gel electrophoresis, showing approximately fragments of 1000 bp and 1200 bp, respectively (Figure 3c); this indicated that the products likely contained the viral genome. Positive bacterial colonies were determined via electrophoresis of the extracted plasmid, which shows the predicted length (Figure 3d). These plasmids were further digested with *Bam*HI to determine whether these plasmids bearing adenovirus genomes were generally constructed using a λ-red recombinase system, and they were confirmed to contain the viral genome via restriction analysis (Figure 3e); positive colonies were sequenced via sequence alignment and no mutations were found. These plasmids were linearized with *Pac*I, then the FAdV-4 genome and pShuttle-FAVITR-FR vector were observed via agarose gel electrophoresis (Figure 3f). Finally, we successfully constructed the vectors containing FAdV-4 genome via Gibson Assembly.

### 3.4. Rescue of FAdV-4 Infectious Clones 

**Figure 4:**
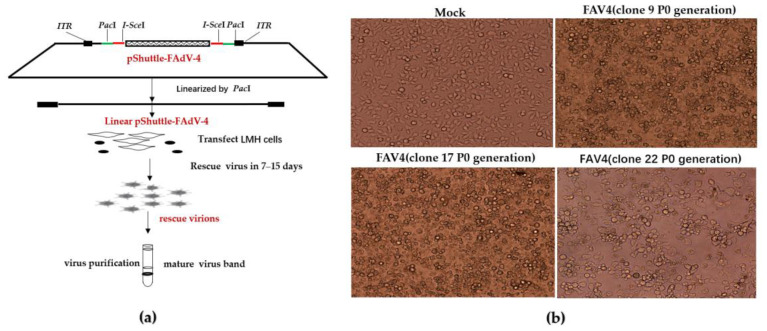
Rescue of FAdV-4 infectious clones. (**a**) Schematic diagram of the rescue of FAdV-4 infectious clone. (**b**) Cytopathic effect of transfected LMH cells.

The virus was rescued from *Pac*I-linearized pShuttle-FAdV-4 transfected LMH cells, as shown in the following diagram (Figure 4a). Fifteen of the eighteen plasmids bearing the Ad genome produced an infectious adenovirus clone and demonstrated a visible CPE and proliferative capacity similar to that of wild-type FAdV-4. The negative control group and partial clones CPE are as follows (Figure 4b): the supernatant in 10 days post-transfection was collected and the virus titers (P0), determined by measuring TCID_50_, ranged from 10^7^ to 10^8^ TCID_50_ (Table A2).

### 3.5. Generation of a Replicating Single-Cycle Adenovirus Vector

**Figure 5:**
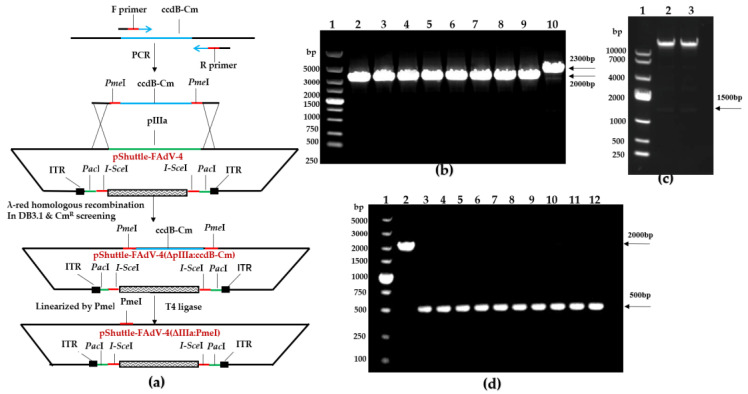
Generation of a single-cycle replicating adenovirus vector. (**a**) Schematic diagram illustrating the construction of the pShuttle-FAdV-4(ΔIIIa:*Pme*I) vector for IIIa gene deletion. (**b**) Identification of positive colonies via colony PCR. Lane 1: DL5000 DNA Marker; Lane 2–9: positive colonies; Lane 10: negative control. (**c**) *Pme*I digestion verification. Lane 1: DL10000 DNA Marker; Lane 2–3: digestion of the pShuttle-FAdV-4(ΔpIIIa:ccdB-Cm)plasmids. (**d**) Identification of positive clones of pShuttle-FAdV-4(ΔIIIa:*Pme*I) by colony PCR. Lane 1: DL5000 DNA Marker; Lane 2: negative control; Lane 3–12: positive colonies.

To construct a replicating single-cycle adenovirus vector, we showed a schematic diagram of how to delete the IIIa gene (Figure 5a). pKD46 was transformed into pShuttle-FAdV-4/DB3.1 competent cells to utilize λ-red homologous recombinase to obtain pKD46 and pShuttle-FAdV-4/DB3.1 cells. These were further transformed with the donor of the whole ccdB cm expression cassette via electroporation to delete the IIIa gene in the FAdV-4 genome. The cells were spread on four agar plates containing kanamycin, ampicillin, and chloramphenicol and incubated at 30 °C overnight. Three hundred colonies grew on each plate, two of which were picked and grown in liquid LB medium containing kanamycin, ampicillin, and chloramphenicol at 30 °C with shaking. Colony PCR was performed with IIIa-up-300-F/IIIa-down-200-R primers, the size of the pShuttle-FAdV4/DB3.1 vector band in the negative control was approximately 2300 bp, and a positive fragment (2000 bp) was observed due to replacement of the IIIa gene with the ccdB cm expression cassette (Figure 5b). The plasmids obtained above were digested by the *Pme*I enzyme, and the target bands of about 1500 bp were digested, which were the same size as the bands of the ccdB cm expression cassette (Figure 5c). These results indicate that the colonies were carrying recombinant Ad vectors in which the IIIa gene was deleted and replaced by the corresponding ccdB cm expression cassette. The pKD46 plasmid could replicate normally at 30 °C, but the thermosensitive replicators could not replicate at 42 °C and could be eliminated by the culture at 42 °C, and the elimination of pKD46 plasmid was verified by checking whether ampicillin resistance existed. The pKD46 eliminated strain was named pShuttle-FAdV-4(ΔIIIa:ccdB-Cm)/DB3.1. To cure the ccdB cm expression cassette, the pShuttle-FAdV-4(ΔpIIIa:ccdB-Cm) plasmid was further linearized by digestion with *Pme*I and used to transform EZ10 competent cells to verify ccdB cm expression cassette deletion. Colony PCR was performed with the primers of IIIa-up-300-F/IIIa-down-200-R, the size of the pShuttle-FAdV-4(ΔpIIIa:ccdB-Cm) vector band in the negative control was 2000 bp, and a positive fragment (500 bp) in pShuttle-FAdV-4(ΔIIIa:*Pme*I) vector was observed due to deletion of the ccdB cm gene (Figure 5d). The ccdB cm expression cassette was successfully removed. 

## 4. Discussion 

The major capsid proteins of FAdV-4 include hexon, penton, and fiber, which make up the majority of the virion surface. The hexon protein contains specific antigenic determinants for adenovirus population, type, and subtype identification, and is currently widely used for virus isolation and identification [38,39]. We isolated a fowl adenovirus that was identified by a phylogenetic tree based on the hexon gene sequence. The fowl adenovirus isolation was classified as serotype FAdV-4 in this study. The virus isolation targets the liver and heart as the main target organs and can cause typical clinical symptoms and pathological changes in animal regression tests. Infected chicks showed 20% mortality, indicating strong virulence of the virus isolation.

Considering the size of the viral genome, the efficiency and convenience of constructing infectious clones remains a challenge. Initially, adenovirus infectious clone construction involves the homologous recombination of a linearized shuttle plasmid carrying a homologous arm with DNA from the adenovirus-containing full-length genome in mammalian cells and screening for plaques containing recombinant adenovirus. This method has major drawbacks due to its low efficiency in homologous recombination and the need for multiple rounds of time-consuming and laborious screening and identification. Later, it was determined that the recA enzyme carried by *E. coli* itself or the introduction of the λ-red recombinase system in *E. coli* can achieve DNA recombination between shuttle plasmids and adenovirus full-length genomes in *E. coli*. Further optimized and improved Gibson Assembly technology can insert approximately 300 kb of target fragments into the target vector. In addition, the SLiCE method and in-fusion technology can also enable recombination between fragments of interest in vitro. In this study, the FAdV-4 vector was initially constructed using in-fusion technology, but the connection efficiency was low. Meanwhile, also in this study, the FAdV-4 vector was successfully constructed via Gibson Assembly, and infectious clones were successfully rescued in LMH cells. The Gibson Assembly method, which is widely used in the construction of infectious clones [40,41,42], has the advantages of convenient operation, simplicity, repeatability, and high efficiency, especially in the construction of adenovirus infectious clones, which has broad application prospects. In addition, TG1 strain was initially used for homologous recombination in this study, but the recombination efficiency was very low and there were few positive clones. Considering the instability of TG1 strain, EZ10 strain was subsequently changed to be more stable for recombination, and a large number of positive clones were obtained. Therefore, when conducting homologous recombination, the selection of more stable recombinant strains would greatly improve the positive efficiency.

Infectious clones can modify or mutate the viral genome, available for fundamental research, gene therapy, and the production of recombinant vaccines [23,25,43,44]. The SC-Ad was created primarily by deleting cement protein IIIa should be taken into consideration as they can replicate the genome and transgenes as well as RC-Ad but do not produce infectious progeny viruses [34,35,45]. Therefore, the safety profile of SC-Ad suggests that these vectors may be useful for vaccines and vector amplification for other therapies. In this study, an RC-Ad vector based on the infectious clone of FAdV-4 was constructed using the λ-red recombineering system. Currently, the world’s leading gene knockout systems include Cre-Loxp, CRISPR/Cas9, and λ-red in vivo homologous recombination technology. We also established the optimal design of paired sgRNAs targeting the IIIa to induce on-target sequence mutation but did not effectively elicit CRISPR/Cas9-mediated cleavage, possibly due to FadV-4 genome insertion in the high-copy vector backbone with the pBR332 ori.

The deficiency of this study is that the deletion of the IIIa gene based on infectious clones requires further virus packaging by complementing the IIIa gene through the cell line. This will be addressed in future work. In the future, we will also study the SC-Ad vector of the FAdV-4 as a vaccine platform.

## 5. Conclusions

In this study, a strain was isolated from a sick flock and identified as FAdV-4 through hexon gene sequencing phylogenetic analysis. Subsequently, infectious clones of FAdV-4 were successfully generated using Gibson Assembly technology and rescued by transfecting LMH cells. In addition, the IIIa gene was deleted based on infectious clones to construct the SC-Ad vector of FAdV-4. 

## Data Availability

Data available on request from the authors.

## Appendix A

**Table A1:** Primers used in the study.

Primer	Sequence (5′-3′)	PCR Product (bp)
hexon-F hexon-R	AACGTCAACCCCTTCAACCACC TTGCCTGTGGCGAAAGGCG	1300
pShuttle-*Pac*I-ITR-F pShuttle-*Pac*I-ITR-R	AAGACGCGGTTATATAAGATGATGTTAATTAAATTAC CCTGTTATCCCTAGAATTAATTCGATCCTGAATGGCG AAGACGCGGTTATATAAGATGATGTTAATTAAATTACC CTGTTATCCCTACATGCATGGATCCATATGCGG	3200
FAdV-4-23500-F FAdV-4-24501-R	TACCAACACGAGCACCACCAAAG GGGTTGACGTTGTCCATCTGGTC	1000
FAdV-4-ITR-F FAdV-4-1176-R	CATCATCTTATATAACCGCGTCTTTTGACACAC GAGCTCCGGTGTAAAGATTCGC	1200
IIIa-up-300-F IIIa-down-200-R	GTGCACGACGGTGGTCGAGC GACCGCCGCTAGAGGAACAG	2300 2000 500

**Table A2:** Virus titer determination.

FAdV-4	CPE Time Point (d)	TCID_50_	
		7 d	10 d
rFAdV-4 (3)	10	10^7.125^	10^7.83^
rFAdV-4 (4)	5	10^7^	10^7.66^
rFAdV-4 (9)	8	10^6.75^	10^7.5^
rFAdV-4 (12)	12	10^6.875^	10^7.3^
rFAdV-4 (15)	16	10^6.75^	10^7.3^
rFAdV-4 (17)	8	10^6.5^	10^7.3^
rFAdV-4 (22)	15	10^6.5^	10^8^
rFAdV-4 (48)	6	10^6.5^	10^7.3^
rFAdV-4 (48)	6	10^6.5^	10^7.3^
rFAdV-4 (53)	8	10^6.625^	10^7.5^
rFAdV-4 (54)	8	10^6.375^	10^7.17^
rFAdV-4 (55)	9	10^6.5^	10^7.5^
rFAdV-4 (57)	8	10^6.75^	10^7.8^
rFAdV-4 (61)	10	10^5.625^	10^7.8^
rFAdV-4 (73)	8	10^6.75^	10^7.5^
